# Exercise mitigates high-fat diet-induced cardiac dysfunction via APOE genotype- and immune-dependent mechanisms: A photon-counting CT study in adult mice

**DOI:** 10.1371/journal.pone.0339293

**Published:** 2025-12-19

**Authors:** Rohan Nadkarni, Alex J. Allphin, Darin P. Clark, Yi Qi, Zay Yar Han, Ketan B. Ghaghada, Alexandra Badea, Cristian T. Badea

**Affiliations:** 1 Quantitative Imaging and Analysis Lab, Department of Radiology, Duke University Medical Center, Durham, North Carolina, United States of America; 2 Department of Radiology, Baylor College of Medicine, Houston, Texas, United States of America; 3 Department of Radiology, Texas Children’s Hospital, Houston, Texas, United States of America; University of Thessaly Faculty of Medicine: Panepistemio Thessalias Tmema Iatrikes, GREECE

## Abstract

**Background:**

Cardiovascular dysfunction frequently accompanies aging and is often worsened by adverse lifestyle factors and genetic susceptibility. The apolipoprotein E (*APOE*) gene modulates susceptibility to cardiovascular disease, but how exercise and diet interact with *APOE* genotype remains insufficiently understood. We investigate the cardioprotective potential of exercise in humanized *APOE*-targeted replacement mice on control and high-fat diet, using photon-counting computed tomography (PCCT) and deep learning-based image segmentation.

**Methods:**

This study included 251 male and female mice in mid-to-late life of *APOE2*, *APOE3*, and *APOE4* genotypes with variation in humanized *NOS2* (*HN*) mediated innate immune response, exercise status (exercised vs. sedentary) and diet (control vs. high-fat). Mice underwent in vivo cine cardiac PCCT imaging following contrast enhancement with liposomal iodine nanoparticles. Stroke volume, ejection fraction, and myocardial mass were derived from automated segmentation of cardiac structures using a 3D U-Net model. We assessed main and interaction effects of genotype, sex, *HN* status, age, exercise and diet using generalized linear models, while Mann-Whitney U tests assessed effects of exercise within stratified subgroups.

**Results:**

Exercise was a significant predictor of improvement in several cardiac functional metrics with a large effect size. The interaction between exercise and diet was a significant predictor of reduced body mass and myocardial mass. Stratified analyses found that exercise improves cardiac functional metrics in *APOE4* mice on both diets, and *APOE3* mice primarily on control diet, while benefitting *HN* mice more than *non-HN* mice.

**Conclusions:**

Voluntary exercise can partially rescue cardiac dysfunction induced by high-fat diet in adult *APOE*-targeted replacement mice, with benefits modulated by genotype, sex, and *HN* status. *APOE4* and *HN* mice benefitted most from exercise. Contrast-enhanced PCCT combined with deep learning segmentation enables scalable, minimally invasive cardiac phenotyping and reveals interaction effects that are critical for designing precision lifestyle interventions in genetically at-risk populations.

## Introduction

Cardiovascular disease (CVD) is a leading cause of morbidity and mortality worldwide, with genetic and lifestyle factors contributing to individual susceptibility. The apolipoprotein E (*APOE*) gene influences lipid metabolism and is known to confer differential risk profiles for CVD across its three major isoforms—*APOE2*, *APOE3*, and *APOE4* [1–3]. While *APOE4* is strongly associated with increased risk for CVD, *APOE2* is thought to confer cardio protection [4,5], though there are also potential vulnerabilities associated as well [6]. Notably, *APOE4* is also the strongest known genetic risk factor for Alzheimer’s disease (AD), reflecting shared vascular and metabolic pathways that influence both heart and brain health. Evolutionary perspectives suggest that high levels of physical activity in ancestral human populations may have mitigated the deleterious cardiovascular and neurodegenerative effects associated with *APOE4*, thereby supporting longer lifespans [7]. This genotype-dependent vulnerability underscores the importance of developing precision approaches in prevention and intervention strategies for CVD, which in turn may have broader implications for treatment of AD.

High-fat diet (HFD) induces obesity, which accelerates cardiac aging and impairs cardiac function [8,9]. Importantly, midlife adiposity also predicts Alzheimer’s disease [10]. How genetic background interacts with environmental factors to compound risk is not fully understood. The *APOE4* genotype leads to a high risk of cardiovascular disease, Alzheimer’s disease, and increased mortality [7]. *APOE* is involved in lipid metabolism, vascular function, and systemic inflammation, and *APOE4* appears to increase cardiovascular risk in humans, particularly under stressors such as HFD [11]. Physical activity is a potent modulator of cardiovascular health and may offset diet- and genotype-related vulnerabilities [12–14]. We hypothesized that voluntary exercise will mitigate HFD-induced cardiac dysfunction in an *APOE* genotype– and sex-dependent manner. We predicted that *APOE4* carriers will show greater HFD-induced cardiac impairment than *APOE3* counterparts; that exercise will attenuate HFD-induced cardiac dysfunction, with a more pronounced benefit in *APOE4* mice; and that the magnitude of diet and exercise effects will reflect known sex-specific responses in cardiovascular physiology [15].

Studies using homozygous humanized *APOE*-targeted replacement mice are useful for testing these hypotheses because these studies are not limited by naturally occurring frequencies of *APOE* alleles and they allow us to tightly control genetic background and lifestyle factors such as exercise and diet. However, most prior studies have not utilized *APOE* mice with a humanized nitric oxide synthase 2 (human *NOS2* or *HN*) background to account for modulation of exercise and diet effects by immune signaling pathways [16]. In this study, we use contrast-enhanced photon-counting CT imaging of homozygous humanized *APOE*-targeted replacement mice for cardiac phenotyping to assess the impact of *APOE* genotype, sex, and *HN*-mediated innate immune response in regulating combined exercise and diet effects on cardiac function.

Advances in CT imaging technology, particularly photon-counting CT (PCCT), have opened new avenues for high-resolution, multi-energy cardiac phenotyping. PCCT provides superior spatial resolution and spectral sensitivity and reduced electronic noise compared to conventional CT [17]. Its utility has been demonstrated in clinical cardiovascular applications such as coronary calcium scoring [18], stent evaluation [19], and myocardial perfusion imaging [20]. In preclinical research, PCCT offers unique advantages for studying the interplay between genotype, environment, and intervention, due to its ability to noninvasively resolve detailed anatomical and functional features [21–23].

Our group has previously used PCCT in two different CVD studies in humanized *APOE* mice. Our initial study comparing sedentary mice on a control diet to sedentary mice on a high-fat diet revealed genotype-specific susceptibilities [24]. In a follow-up study, which examined the effects of exercise alone by including sedentary mice on control diet and exercised mice on control diet, we demonstrated sex- and genotype-dependent benefits of voluntary exercise [25]. However, neither study addressed how diet and exercise interact, nor whether different combinations of these lifestyle factors modify cardiac aging differently depending on genotype, sex, or immune background.

The present study builds on our prior studies to test our predictions regarding the modulation of exercise/diet interaction effects by *APOE* genotype, sex, and *HN* immune status. We include mice from the three major homozygous *APOE* genotypes, both sexes, with/without humanized *NOS2* (*HN*) background, and exercise vs. sedentary conditions, under both control diet and HFD regimens. Using high-resolution, PCCT-based cardiac phenotyping of these mice, we aim to evaluate how exercise mitigates HFD-induced cardiac dysfunction in a genotype-, sex-, and immune background–dependent manner. By integrating two major and opposing lifestyle factors, our work aims to provide new insights into how modifiable (diet, exercise) and non-modifiable (genotype, sex, immune status) factors jointly shape cardiovascular outcomes, advancing the goal of developing precision lifestyle interventions for at-risk populations.

## Materials and methods

### Mouse models and study design

All procedures were conducted in accordance with NIH guidelines and approved by the Duke University Institutional Animal Care and Use Committee (IACUC protocol A173-20–08). Following completion of the study, the mice were euthanized using an intraperitoneal injection of 250 mg/Kg pentobarbital, as approved by our institution’s animal care and use committee. We ensured that all actions were carried out humanely and with the utmost concern for the welfare of our animals. We used a cohort of 251 mice including male and female mice that are homozygous for one of the 3 major human *APOE* alleles (*APOE2*, *APOE3*, *APOE4*), with a subset carrying a humanized immune background through insertion of the human *NOS2* gene [16,26,27], henceforth referred to as the *HN* factor. Given that the *NOS2* gene codes for the inducible NOS (iNOS) protein that produces nitric oxide (NO) in immune cells such as macrophages and astrocytes [27], replacement of the mouse *Nos2* gene with the human *NOS2* gene in *HN* mice makes their immune response more like that of humans [16,26]. The age of the mice at the time of imaging was 14.2 ± 3.1 months, corresponding to mid-to-late life in the murine lifespan [28]. Mice were assigned to one of four groups in a 2 × 2 factorial layout: (1) control diet with sedentary housing, (2) control diet with exercise, (3) high-fat diet with sedentary housing, or (4) high-fat diet with exercise. To isolate the effects of exercise and dietary manipulation, mouse cages were randomly assigned to the groups across genotypes and sexes. Distribution across experimental subgroups is detailed in Table 1. All mice were housed under reverse light-dark cycles for the duration of the interventions, and had ad libitum access to water and their respective diets.

**Table 1:** Number of mice grouped by *APOE* genotype, sex, diet, exercise participation, and HN status (total 251 mice).

Genotype	Sex	Diet	Exercise	Count	%*HN*
*APOE2*	Female	CTRL	Yes	20	45.00% (9/20)
			No	7	42.86% (3/7)
		HFD	Yes	9	44.44% (4/9)
			No	7	42.86% (3/7)
	Male	CTRL	Yes	18	44.44% (8/18)
			No	9	33.33% (3/9)
		HFD	Yes	11	54.55% (6/11)
			No	8	37.50% (3/8)
*APOE3*	Female	CTRL	Yes	15	53.33% (8/15)
			No	8	37.50% (3/8)
		HFD	Yes	8	62.50% (5/8)
			No	10	70.00% (7/10)
	Male	CTRL	Yes	16	50.00% (8/16)
			No	6	50.00% (3/6)
		HFD	Yes	9	44.44% (4/9)
			No	14	50.00% (7/14)
*APOE4*	Female	CTRL	Yes	12	41.67% (5/12)
			No	7	57.14% (4/7)
		HFD	Yes	10	60.00% (6/10)
			No	7	71.43% (5/7)
	Male	CTRL	Yes	12	33.33% (4/12)
			No	10	70.00% (7/10)
		HFD	Yes	7	71.43% (5/7)
			No	11	36.36% (4/11)

### Diet

Two dietary regimens were used: a control diet (CTRL) and a high-fat diet (HFD). The control diet was a standard low-fat chow (LabDiet2001), formulated to maintain healthy metabolic profiles in aging mice. The high-fat diet (Research Diets D12492) consisted of 60% kcal from fat, 20% from protein, and 20% from carbohydrates, designed to model a Western-style diet that induces metabolic stress. This HFD model has been previously used in our studies in adult mice [24]. Mice maintained on HFD consumed an average of 3.02 ± 0.44 grams of food per day, consistent with previously reported intake ranges for rodents on energy-dense diets [29]. This intake is lower than that of mice fed CTRL (4.31 ± 0.67 grams), reflecting the expected downregulation in gram-based consumption when mice are fed HFD due to its higher caloric density [30]. Both diets were provided ad libitum for a minimum of 12 weeks prior to imaging, allowing sufficient time for cardiac and systemic physiological adaptation.

### Exercise

Exercised mice were provided access to individual running wheels (Med Associates, Inc., St. Albans, VT, USA) for 1 hour per day, 5 days per week, over a 3-month period.

### Cardiac imaging and analysis pipeline

**Fig 1:**
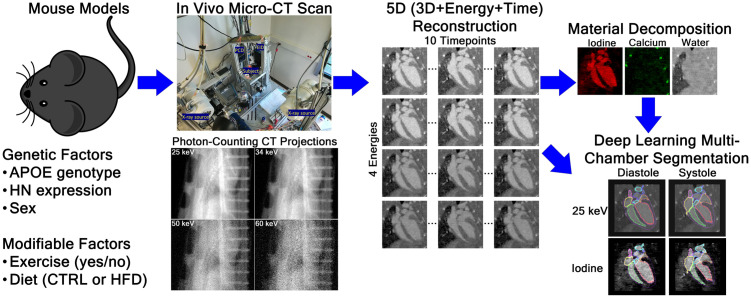
Flowchart for our cardiac imaging and analysis pipeline.

Fig 1 summarizes our cardiac imaging and analysis pipeline, which was developed in our prior work [25]. In vivo cardiac imaging was performed using our custom-built PCCT system equipped with a Varian G297 x-ray tube and a Dectris Santis 1604 CdTe detector with four energy thresholds and a 150 µm pixel size [31]. Each mouse received a retro-orbital injection of liposomal iodine nanoparticle contrast agent (Lip-I) and was scanned using settings of 80 kVp, 4 mA, and 10 ms per projection, with 7000 projections acquired over 1070 degrees rotation and 12.5 mm vertical translation, and energy thresholds set to 25, 34, 50, and 60 keV. While the protocol included a brief intravenous injection of contrast agent, no surgical or catheter-based procedures were performed, and the imaging component of the protocol is non-invasive in nature. As described previously, mice were anesthetized during the PCCT scan using 2–3% isoflurane delivered through a nosecone, breathing was monitored using a pillow with a pressure transducer, and ECG was monitored using electrodes placed on the paws of the mouse [24]. Projections were sorted into ten cardiac phases using an intrinsic gating approach described previously [24] and reconstructed using a multi-channel iterative algorithm with a uniform voxel resolution of 125 µm. This reconstruction incorporated joint regularization across spectral and temporal domains via rank-sparse kernel regression (RSKR) [32,33].

The reconstructed PCCT images were decomposed into material maps (e.g., iodine, photoelectric effect, and Compton scattering; or iodine, calcium, and water) using a variation of the method described by Alvarez and Macovski [32,34], with orthogonal subspace projection to enforce non-negative material concentrations [32].

Segmentations of the atria, ventricles, myocardium, aorta, and pulmonary artery across diastolic and systolic phases were generated using our 3D U-Net models that were previously trained with manually labeled datasets [25]. Although we have trained two segmentation models, one with CT images at the first energy threshold as input and another with iodine material maps as the input, only segmentations from the model with CT images as input were used in the current study because this model was found to have superior quantitative accuracy [25].

From these segmentations, we computed a set of physiological and cardiac metrics: stroke volume (SV), ejection fraction (EF), and myocardial mass (MM).

SV was calculated as the difference between end-diastolic volume (EDV) and end-systolic volume (ESV) of the left ventricle:


$$\begin{array}{c} SV=EDV-ESV\; \end{array}$$ (1)

EF, representing the percentage of blood ejected from the left ventricle, was calculated as:


$$\begin{array}{c} EF(\%)=\frac{SV}{EDV}\times \text{1}\text{0}\text{0} \end{array}$$ (2)

Right ventricle (RV) stroke volume was calculated as shown in Equation 1 using the EDV and ESV of the RV. Myocardial volume was approximated using the average volume of the segmented myocardium across both the diastolic and systolic phases. This myocardial volume was converted to MM using an assumed tissue density of 1.053 g/mL [35].

### Data analysis and statistical modeling

Six primary physiological and cardiac metrics were assessed: body mass of the mouse, heart rate (HR), SV, EF, RV SV, and MM. Although there is a statistically significant, moderately strong positive linear relationship between SV and EF in our entire cohort and in sex-specific subgroups (S1 Table), these metrics capture different physiological information. SV measures the absolute blood volume ejected per beat, while EF measures contractile efficiency relative to the EDV. Due to substantial variation in EDV in our cohort (mean = 0.055 mL, range = 0.089 mL), it is possible for two mice to have similar SV but very different EF (if EDV differs), or conversely, very different SV but similar EF (if SV and EDV scale proportionally). Therefore, it was necessary to retain both SV and EF as dependent variables in our study despite their correlation.

To evaluate the influence of modifiable factors such as diet and exercise and biological factors such as *APOE* genotype, sex, and immune background (*HN* status), we implemented a multi-step statistical workflow comprising model assumption checks, multi-factorial analysis, and stratified subgroup comparisons.

### Assumption testing

Before statistical modeling, we assessed whether each physiological or cardiac metric met the assumptions of normality and homogeneity of variance. Normality was tested using the Shapiro-Wilk test [36], and homogeneity of variance across experimental subgroups was evaluated using Levene’s test [37]. Table 2 summarizes the resulting p-values for each metric. For both tests, a p-value less than 0.05 indicates rejection of the assumption.

**Table 2:** P-values from Shapiro-Wilk and Levene’s test for each metric.

Metric	Shapiro p-value	Levene p-value
Mass	6.13 × 10^−4^	0.0423
Heart Rate	0.524	0.966
Stroke Volume	0.911	0.659
Ejection Fraction	0.142	0.688
RV Stroke Volume	0.605	0.692
Myocardial Mass	5.20 × 10^−5^	0.981

Metrics that satisfied both normality and homogeneity of variance assumptions (HR, SV, EF, and RV SV), were analyzed using generalized linear models (GLMs) with a Gaussian distribution and identity link function. To provide a visual representation of the effect of each of our predictors on cardiac function, we ran analysis of variance (ANOVA) on the GLMs for these four cardiac metrics. From this ANOVA, we plotted a heatmap showing the effect size of each predictor on each cardiac metric. Effect sizes were reported using partial eta-squared (η²), which represents the proportion of variance in the dependent variable explained by each factor after controlling for other terms in the model [38]. Due to the correlation between SV and EF, significant results from the GLMs for these two metrics are not independent of each other. Therefore, we also ran a multivariate analysis of variance (MANOVA) with both SV and EF as dependent variables.

MM, which violated the assumption of normality, and body mass, which failed both normality and homogeneity of variance tests, were analyzed using GLMs with a Gamma distribution and log link function, consistent with statistical recommendations for skewed continuous physiological data [39].

### Multi-factor statistical analyses

For all GLMs, we used a comprehensive interaction model with two-way interactions and the following structure:

$\begin{array}{c} VAR\;\sim \;Genotype\;+\;Sex\;+\;HN\;+\;Age\;+\;Exercise\;+\;Diet\;+\;Genotype\;:\;Sex\;+\;Genotype\;:\;HN\;\\ +\;Genotype\;:\;Exercise\;+\;Genotype\;:\;Diet\;+\;Sex\;:\;HN\;+\;Sex\;:\;Exercise\;+\;Sex\;:\;Diet\;+HN\;:\;Exercise\;\\ +\;HN\;:\;Diet\;+\;Exercise\;:\;Diet \end{array}$

Where VAR refers to one of our 6 physiological or cardiac metrics, Genotype refers to one of the 3 homozygous *APOE* genotypes without considering *HN* (*APOE2*, *APOE3*, or *APOE4*), and *HN* refers to presence or absence of a humanized immune background without considering *APOE* genotype (*HN* or *non-HN*). Our models use exercise as a categorical variable because we did not consistently measure the distance run for all 147 exercised mice in our cardiac PCCT study. However, S2 Table reports the mean and 95% confidence interval (CI) of distance run (in km) grouped by *APOE* genotype alone, *HN* status alone, and diet alone for a group of 152 mice that includes a subset of mice from this PCCT study as well as mice used in other studies. Our GLM formula accounts for the main effect of 5 categorical predictors and 1 continuous predictor (age in months) as well as the effect of 2-way interactions between categorical predictors on our metrics. For all our GLMs, the reference (level 0) values of the categorical variables in our model were male, *APOE3*, *non-HN*, no exercise, and CTRL diet.

For MANOVA, the formula was the same as the univariate GLM formula shown above, except that VAR now refers to the joint/multivariate SV and EF outcomes. For each predictor we computed the Wilks’ λ and Pillai’s trace as well as the p-values associated with using each one as the test statistic. We considered a predictor to be statistically significant if both p-values were below the 5% threshold. Since we only ran MANOVA for one pair of cardiac metrics (SV and EF), we did not adjust its p-values for multiple comparisons.

### Stratified subgroup comparisons

To further explore context-dependent effects of exercise, we conducted Mann-Whitney U tests within subgroups defined by diet alone or combinations of diet and one biological factor (genotype, sex, or *HN* status). For example, we compared the values of our metrics from exercised vs. sedentary mice within subgroups such as *APOE3*-HFD, Female-CTRL, or *HN*-HFD. This approach allowed us to detect differential responsiveness to exercise within biologically meaningful strata. We chose a non-parametric test because normality cannot be assumed due to the small sample sizes of these strata.

### Statistical prioritization and multiple comparisons control

Given the large number of potential comparisons across genotype, sex, diet, exercise, and *HN* status, we implemented a structured approach to reduce the risk of inflated Type I error and to focus on biologically relevant effects. Prior to analysis, we identified **Genotype:Exercise** and **HN:Exercise** as our primary interactions of interest and gave secondary consideration to **sex-specific differences**. These interactions and their associated main effects and relevant stratified analyses were interpreted in the context of the study hypotheses. Analyses involving other effects were considered exploratory and are presented in the *Supplementary information* section. For all GLMs and Mann-Whitney U tests, p-values were adjusted for multiple testing using the Benjamini–Hochberg false discovery rate (FDR) procedure [40]. During FDR correction, p-values were grouped by predictor across all relevant metrics. Effect sizes (η² or partial η²) and coefficients from our GLMs (β) were reported alongside p-values to emphasize magnitude and direction of effects. This approach ensures that statistical interpretation is guided by hypothesis-driven priorities while maintaining transparency of the full dataset.

### Software and implementation

All statistical analyses were performed in Python, using the *SciPy, statsmodels, pandas, and seaborn* packages. A significance threshold of p < 0.05 was applied throughout.

## Results

We computed the mean and 95% CI for each metric in groups based on unique combinations of sex, *APOE* genotype, exercise status, and diet. These results are provided in S3 Table.

### Multi-factor statistical analyses

Out of the 27 significant predictors across our 6 GLMs, 17 involved main or interaction effects of exercise or diet. These 17 predictors are summarized in Table 3. The remaining 10 significant predictors are listed in S4 Table. Our MANOVA with SV and EF as dependent variables returned 5 significant predictors, which are summarized in S5 Table.

**Table 3:** Summary of significant exercise and diet effects in our GLMs.

Metric	Predictor	Coefficient (β)	Corrected p-value	Interpretation
Mass	Diet[T.HFD]	0.364	<10^−7^	High-fat diet has a significant positive effect on body mass relative to control diet.
Mass	Sex[T.Female]: Exercise[T.Exercise]	−0.118	3.28 × 10^−3^	The combination of female sex and exercise has a significant negative effect on body mass.
Mass	Exercise[T.Exercise]: Diet[T.HFD]	−0.175	1.42 × 10^−5^	The combination of exercise and diet has a significant negative effect on body mass.
Stroke Volume	Exercise[T.Exercise]	5.31 × 10^−3^	0.0348	Exercise has a significant positive effect on stroke volume relative to no exercise.
Stroke Volume	Geno3[T.APOE4]: Diet[T.HFD]	6.86 × 10^−3^	3.09 × 10^−3^	The combination of *APOE4* and high-fat diet has a significant positive effect on stroke volume.
Stroke Volume	Sex[T.Female]: Exercise[T.Exercise]	−5.07 × 10^−3^	7.54 × 10^−3^	The combination of female sex and exercise has a significant negative effect on stroke volume.
Stroke Volume	HN[T.HN]: Diet[T.HFD]	4.75 × 10^−3^	0.0166	The combination of *HN* and high-fat diet has a significant positive effect on stroke volume.
Ejection Fraction	Exercise[T.Exercise]	7.90	0.0348	Exercise has a significant positive effect on ejection fraction.
Ejection Fraction	HN[T.HN]: Diet[T.HFD]	6.27	0.0207	The combination of *HN* and high-fat diet has a significant positive effect on ejection fraction.
RV Stroke Volume	Exercise[T.Exercise]	4.66 × 10^−3^	0.0427	Exercise has a significant positive effect on RV stroke volume relative to non-exercise.
RV Stroke Volume	Geno3[T.APOE4]: Diet[T.HFD]	6.72 × 10^−3^	3.09 × 10^−3^	The combination of *APOE4* genotype and high-fat diet has a significant positive effect on RV stroke volume.
RV Stroke Volume	Sex[T.Female]: Exercise[T.Exercise]	−4.37 × 10^−3^	0.0136	The combination of female sex and exercise has a significant negative effect on RV stroke volume.
RV Stroke Volume	HN[T.HN]: Diet[T.HFD]	5.25 × 10^−3^	8.85 × 10^−3^	The combination of *HN* and high-fat diet has a significant positive effect on RV stroke volume.
Myocardial Mass	Geno3[T.APOE4]: Exercise[T.Exercise]	0.134	0.0412	The combination of *APOE4* genotype and exercise has a significant positive effect on myocardial mass.
Myocardial Mass	Geno3[T.APOE4]: Diet[T.HFD]	0.149	4.98 × 10^−3^	The combination of *APOE4* genotype and high-fat diet has a significant positive effect on myocardial mass.
Myocardial Mass	HN[T.HN]: Exercise[T.Exercise]	0.120054	0.0176	The combination of *HN* and exercise has a significant positive effect on myocardial mass.
Myocardial Mass	Exercise[T.Exercise]:Diet[T.HFD]	−0.117	0.0192	The combination of exercise and high-fat diet has a significant negative effect on myocardial mass.

Our results are consistent with the expectation that exercise improves cardiac function, with the main effect of exercise found to be a statistically significant predictor with a positive coefficient for SV (β = 5.31 × 10^−3^, p = 0.0348), EF (β = 7.90, p = 0.0348), and RV SV (β = 4.66 × 10^−3^, p = 0.0427). The main effect of high-fat diet was found to be a significant predictor of increased body mass (β = 0.364, p < 10^−7^) but was not a significant predictor of any other cardiac metric. The interaction between exercise and diet (Exercise[T.Exercise]:Diet[T.HFD]) appeared as a significant predictor twice, implying that exercise counteracts high-fat diet induced increases in both body mass (β = −0.175, p = 1.42 × 10^−5^) and myocardial mass (β = −0.117, p = 0.0192).

The interaction between sex and exercise (Sex[T.Female]:Exercise[T.Exercise]) was a significant predictor of several cardiac metrics, having a negative effect on body mass (β = −0.118, p = 3.28 × 10^−3^), SV (β = −5.07 × 10^−3^, p = 7.54 × 10^−3^), and RV SV (β = −4.37 × 10^−3^, p = 0.0136). This suggests that compared to exercised males, exercised females experience greater reduction in body mass but less improvement in cardiac function (in terms of blood volume ejected per beat).

The interaction between HN and diet (HN[T.HN]:Diet[T.HFD]) had a significant positive effect on SV (β = 4.75 × 10^−3^, p = 0.0166), EF (β = 6.27, p = 0.0207), and RV SV (β = 5.25 × 10^−3^, p = 8.85 × 10^−3^), implying that the HN gene mitigates high-fat diet induced impairment of cardiac function.

We found several significant interaction effects between APOE genotype and diet (Geno3[T.APOE4]:Diet[T.HFD]) and between APOE genotype and exercise (Geno3[T.APOE4]:Exercise[T.Exercise]). The interactions with diet suggest that compared to the APOE3 genotype, the APOE4 genotype has more of a tendency to counteract high-fat diet induced impairment of SV (β = 6.86 × 10^−3^, p = 3.09 × 10^−3^) and RV SV (β = 6.72 × 10^−3^, p = 3.09 × 10^−3^), but is less resistant to an increase in myocardial mass due to high-fat diet (β = 0.149, p = 4.98 × 10^−3^). The interaction with exercise suggests that compared to APOE3, APOE4 is less likely to have reduced myocardial mass when exercised (β = 0.134, p = 0.0412).

**Fig 2:**
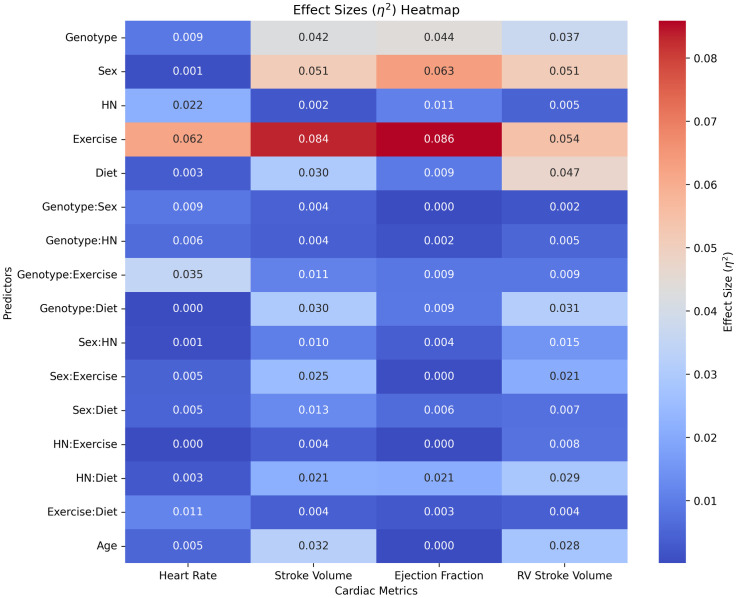
Heatmap showing effect size (η²) of each predictor on cardiac metrics.

Fig 2 shows a heatmap of the effect size of predictors in our ANOVA for HR, SV, EF, and RV SV. Based on the observed range of values, we consider an effect size between 0 and 0.03 to be small, 0.03 to 0.06 to be medium, and 0.06 and above to be large. The main effect of exercise had a medium to large effect on all four cardiac metrics, while the main effect of diet had a medium effect on SV and RV SV. This analysis found the interaction between exercise and diet to have only a small effect on all four metrics, which may indicate that the effect of this interaction varies dramatically based on genetic (non-modifiable) factors.

Among non-modifiable factors, we found the main effect of sex to have medium to large effects and the main effect of genotype to have medium effects on SV, EF, and RV SV. The main effect of age had small to medium effects on SV and RV SV. Although effect sizes of interaction predictors were relatively small, some of the interactions that had small to medium effects (η² > 0.02) on multiple cardiac metrics include Genotype:Diet, Sex:Exercise, and HN:Diet.

Overall, these findings reinforce the value of exercise as a modifiable intervention that can improve cardiac function, even under metabolic stress. They also highlight the importance of considering *APOE* genotype and *HN* status when modeling cardiovascular risk and response. This work supports a stratified approach to preclinical cardiometabolic research that accounts for complex gene–environment–lifestyle interactions.

### Stratified subgroup comparisons

**Fig 3:**
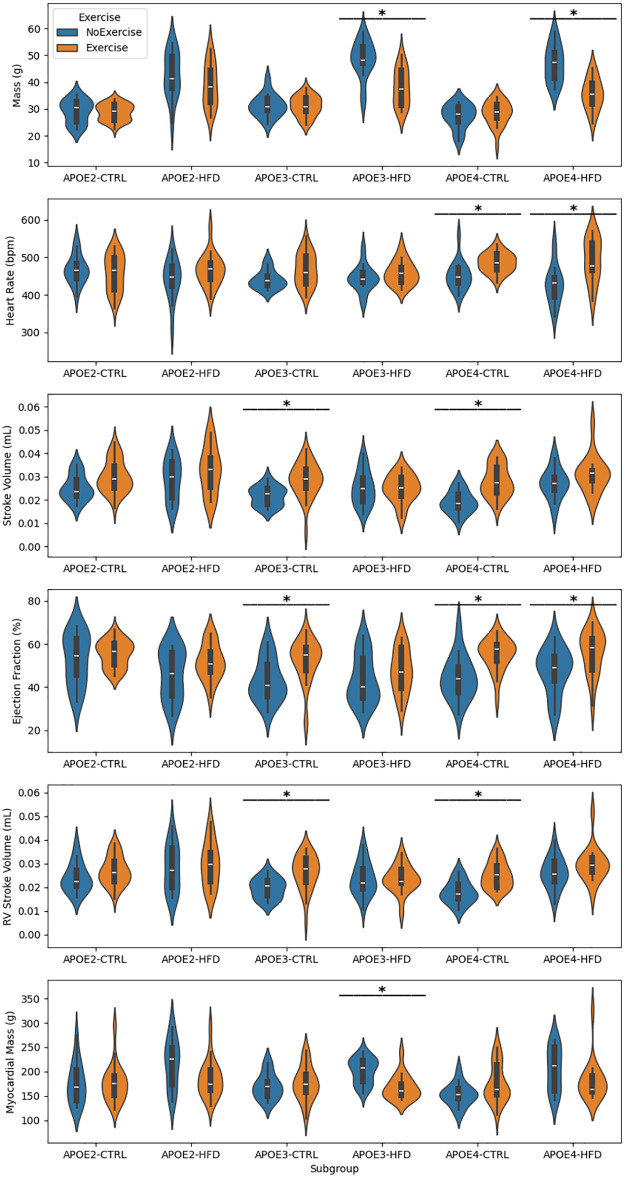
Effects of exercise on cardiac metrics across *APOE* genotype and diet subgroups. Violin plots show six physiological and cardiac metrics stratified by *APOE* genotype (*APOE2*, *APOE3*, *APOE4*), diet (CTRL vs. HFD), and exercise status. Boxplots within violins show the median and interquartile range. Asterisks denote significant differences (p < 0.05, Mann-Whitney U test) by exercise status within a genotype-diet subgroup.

**Fig 4:**
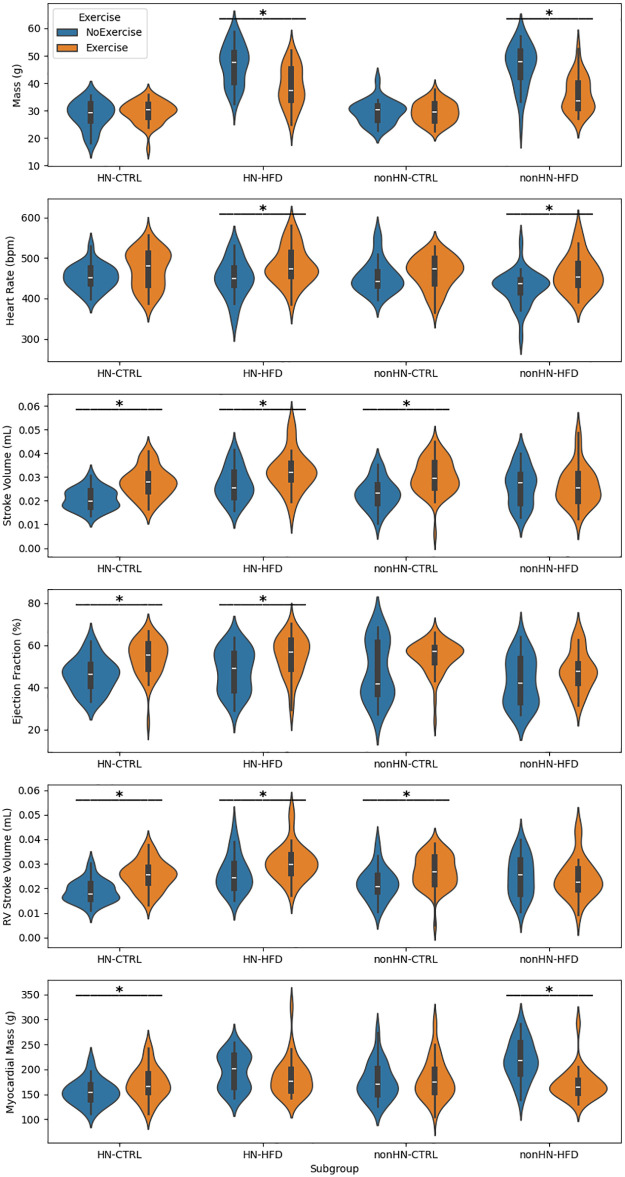
Effects of exercise on cardiac metrics across *HN* status and diet subgroups. Violin plots show six physiological and cardiac metrics stratified by *HN* status (*HN* vs. *non-HN*), diet (CTRL vs. HFD), and exercise status. Boxplots within violins show the median and interquartile range. Asterisks denote significant differences (p < 0.05, Mann-Whitney U test) by exercise status within a *HN* status-diet subgroup.

Our violin plots showing responses to exercise within diet subgroups and within sex-by-diet subgroups are in S1 Fig and S2 Fig, respectively. Our genotype-by-diet and *HN* status-by-diet stratified analyses, which are most crucial for evaluation of our study hypotheses, are in Figs 3-4.

Fig 3 illustrates genotype-specific responses to exercise across six physiological and cardiac metrics under both CTRL and HFD conditions. Within the CTRL group, *APOE3* and *APOE4* mice exhibited robust improvements in SV, EF, and RV SV following exercise. These improvements are evidenced by consistently higher medians in the exercised cohorts compared to sedentary counterparts. Under HFD conditions, *APOE4* mice continued to show significant improvement in EF from exercise, suggesting partial preservation of cardioprotective effects despite dietary stress. In contrast, *APOE3*-HFD did not show significant improvement in SV, EF, or RV SV with exercise. However, both *APOE3*-HFD and *APOE4*-HFD mice show significant reduction in mass with exercise. Notably, *APOE2* mice demonstrated no significant exercise-induced improvements in any measured physiological or cardiac metric under either dietary condition. Collectively, these findings highlight a strong genotype-exercise interaction effect on cardiac function. Physical activity was more likely to produce significant cardioprotective effects on metrics in *APOE3* and *APOE4* mice, whereas *APOE2* mice remained largely refractory, underscoring the importance of genetic background in determining cardiovascular adaptability to lifestyle interventions.

Fig 4 examines the effect of exercise in groups stratified by *HN* status and diet. Mice expressing the *HN* gene exhibited more consistent and robust improvements in cardiac function following exercise, compared to their *non-HN* counterparts.

In *HN* mice, exercise significantly increased SV, EF, and RV SV under both CTRL and HFD conditions. In contrast, *non-HN* mice showed a more variable and attenuated response to exercise. Although some improvements in functional metrics such as SV and RV SV were observed under CTRL diet, there was no statistically significant effect of exercise on these metrics under HFD. This suggests that the absence of humanized *NOS2* may limit the physiological adaptability of the heart to exercise under metabolic stress. However, exercise did significantly reduce body mass in both *HN* and *non-HN* mice under HFD, indicating a protective effect against diet-induced systemic weight gain that does not depend on *HN* status.

These findings support the hypothesis that *HN* expression enhances cardiovascular resilience, enabling more effective responses to lifestyle interventions such as exercise. The differential effects observed between *HN* and *non-HN* groups underscore the importance of immune signaling pathways in modulating the cardiac response to external stressors, and highlight the broader interplay between genotype, immune status, diet, and physical activity in shaping age-related cardiovascular outcomes.

### Qualitative assessment

**Fig 5:**
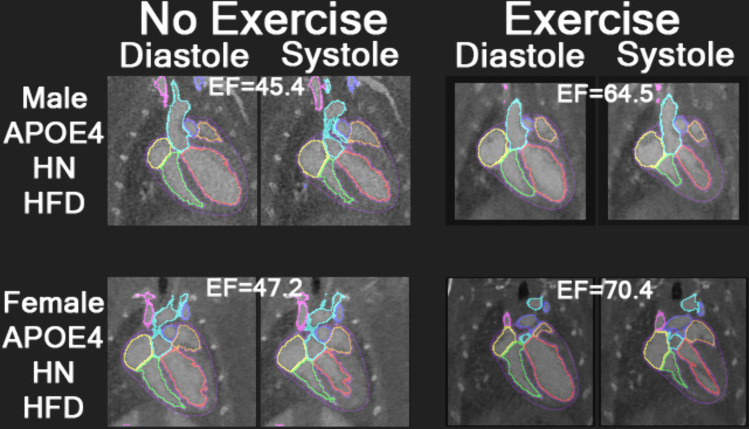
Qualitative comparison of multi-chamber heart segmentations from example PCCT images of *APOE4*-*HN*-HFD mice of both sexes and both exercise regimens. For each of the 4 mice, PCCT images with overlayed heart chamber segmentations are shown at both the diastole and systole cardiac phases. The EF value for each mouse is overlayed on its corresponding images.

Fig 5 provides a qualitative comparison of representative PCCT images from male and female *APOE4*-*HN*-HFD mice both with and without exercise. Segmented cardiac chambers (e.g., left/right ventricles and atria) are overlaid to illustrate functional differences between exercise conditions. Within each sex, the exercised mouse shows noticeably smaller ventricular volumes at systole than the non-exercised mouse, indicative of more effective contraction and higher ejection fraction. These visual findings align with the quantitative data from the broader cohort, which demonstrated significant exercise-induced improvements in EF and other cardiac functional metrics. While qualitative in nature, these examples visually reinforce the cardioprotective effects of exercise, particularly in the context of *APOE4* genotype, *HN* immune background, and dietary stress.

## Discussion

Our results demonstrate that voluntary exercise effectively mitigates cardiac dysfunction induced by a high-fat diet in mice with humanized *APOE* genotypes, which model different degrees of CVD and neurodegenerative disease risk [41]. The cardioprotective effects of exercise were modulated by genotype and immune background (*HN* status). By leveraging high-resolution photon-counting CT and automated deep learning segmentation, we achieved high resolution, minimally invasive phenotyping of whole-heart function in a large, biologically diverse mouse cohort. Our results extend previous work [24,25] by revealing complex, context-specific interaction effects that shape cardiovascular outcomes in aging.

### Cardioprotective effects of exercise and their modifiers

Exercise improved cardiac function across multiple metrics, including SV, EF, and RV SV, in several genotype–diet contexts, with the magnitude and consistency of benefit varying across *APOE* genotype, immune background, and dietary condition. These findings confirm exercise as a dominant and reproducible modulator of cardiovascular performance in aging [42]. However, we found that exercise boosts inotropy (increases SV, EF) in a genotype-specific pattern: *APOE2* do not show significant gains; *APOE3* show improvement in SV, EF, and RV SV on CTRL but no improvement on HFD; *APOE4* exhibit improvements in all three of these functional metrics on CTRL as well as improvement in EF on HFD. For HFD mice, exercise reduces body mass in the *APOE3* and *APOE4* genotypes but not *APOE2*. Chronotropy is mostly unchanged—except in *APOE4*, which alone shows increased HR with exercise. In short, running induces a stronger heart in *APOE3/4* and selectively reverses HFD-induced remodeling in *APOE4*. This mirrors human studies suggesting that individuals carrying the *APOE4* allele, while at higher risk for CVD, may exhibit enhanced responsiveness to lifestyle interventions [13,14]

Immune background further modulated the response to exercise. *HN*-positive mice showed more consistent improvements in cardiac function (e.g., SV, EF, RV SV) with exercise under both diets. However, reduction in myocardial mass under HFD was only statistically significant in *non-HN* mice, suggesting that immunomodulatory effects may not uniformly protect against structural remodeling. These findings support a role for immune signaling in shaping cardiac adaptability but also highlight the complexity of immune background by environment interactions.

### Implications for translational research

This study reinforces the potential of targeted exercise regimens as non-pharmacological interventions for genetically at-risk populations. Our data suggest that precision lifestyle intervention tailored to genotype is a promising approach for mitigating cardiovascular decline in aging. In humans, prior studies have shown that while *APOE4* carriers are at elevated baseline risk for cardiovascular and neurodegenerative disease, they often experience disproportionately greater improvements in aerobic fitness, vascular function, or cardiometabolic risk markers following structured exercise compared with non-carriers, particularly under adverse metabolic conditions [7,13,14]. The genotype- and diet-dependent benefits we observed in *APOE3* and *APOE4* mice—together with the blunted response in *APOE2*—mirror these human patterns and support the translational relevance of our model. Our findings suggest that *APOE4* carriers and individuals with heightened iNOS-mediated immune responses may be ideal candidates for more aggressive lifestyle-based prevention strategies. The modulating role of the *HN* immune background in our murine model suggests that immune signaling pathways—such as those involving *NOS2*—may influence individual variability in exercise responsiveness and merit further investigation in humans. This raises the possibility of developing immune-related biomarkers to guide personalized exercise prescriptions in clinical populations. However, it is important to note that murine physiology, diet composition, and activity patterns differ from those in humans. Direct translation will require studies in diverse human cohorts that assess analogous imaging biomarkers and examine whether similar patterns of diet–exercise interaction to those we found in mice occur in humans.

### Limitations

While the findings of this study are robust and informative, several limitations should be acknowledged. First, the cross-sectional design limits our ability to assess the long-term durability of exercise-induced cardiac benefits. A longitudinal approach would provide valuable insight into how cardiac function evolves with continued physical activity. Additionally, the study did not include behavioral or metabolic assessments, such as VO₂ max testing or glucose tolerance, which restricts the interpretation of cardiac improvements in relation to overall physiological performance.

Moreover, the absence of molecular or cellular characterization limits mechanistic interpretation. Key pathways involved in exercise response, such as nitric oxide signaling, inflammation, and lipid metabolism, remain unexplored in this work and represent important avenues for future investigation.

Finally, it is important to note that species-specific differences between mice and humans may constrain the direct translational relevance of these findings. While murine *APOE* models provide valuable insights into genotype-dependent cardiovascular dynamics, caution is warranted when extrapolating to human physiology.

## Conclusions

Our findings reveal that exercise robustly improves cardiac function in adult mice, even under dietary stress, and that its efficacy is differentially modulated by *APOE* genotype, sex, and immune background. In particular, we found the *APOE4* genotype and *HN* expression to be associated with greater improvements in key functional metrics with exercise under both control and high-fat diet conditions, although the degree of exercise induced remodeling varied by diet. These context-specific responses support the development of personalized lifestyle interventions for aging populations at risk of cardiovascular decline. Moreover, this study highlights the value of PCCT imaging combined with AI-driven analytics for accelerating preclinical cardiovascular research and precision medicine discovery.

## Supporting information

S1 Table Pearson’s correlation between SV and EF in entire mouse cohort and in subgroups by sex. (DOCX)

S2 Table Mean and 95% confidence interval of distance run grouped by *APOE* genotype, *HN* status, and diet. This group of 152 mice includes a subset of those in our cardiac PCCT study as well as mice that were not scanned with PCCT. Grouping in this table is only done by a single categorical variable at a time.
(DOCX)

S3 Table Mean and 95% confidence interval of physiological and cardiac metrics grouped by sex, *APOE* genotype, exercise regimen, and diet plan. Lower and upper bounds of 95% confidence interval are shown in brackets below each mean value.
(DOCX)

S4 Table Summary of significant effects in our GLMs that did not involve exercise or diet. (DOCX)

S5 Table Summary of significant effects in our MANOVA with stroke volume and ejection fraction as dependent variables. For each significant predictor, we report the value of two test statistics (Wilks’ λ and Pillai’s trace), with the associated p-value shown in parentheses.
(DOCX)

S1 Fig Effects of exercise on cardiac metrics across diet subgroups. Violin plots show six physiological and cardiac metrics stratified by diet (CTRL vs. HFD) and exercise status. Boxplots within violins show the median and interquartile range. Asterisks denote significant differences (p < 0.05, Mann-Whitney U test) by exercise status within a diet subgroup.
(TIF)

S2 Fig Effects of exercise on cardiac metrics across sex and diet subgroups. Violin plots show six physiological and cardiac metrics stratified by sex (female/male), diet (CTRL/HFD), and exercise status. Boxplots within violins show the median and interquartile range. Asterisks denote significant differences (p < 0.05, Mann-Whitney U test) by exercise status within a sex-diet subgroup.
(TIF)
