# Supplementary material for: Exercise mitigates high-fat diet-induced cardiac dysfunction via APOE genotype- and immune-dependent mechanisms: A photon-counting CT study in adult mice
Source: PLoS One. 2025 Dec 19;20(12):e0339293. doi: 10.1371/journal.pone.0339293 (PMC12716737; doi:10.1371/journal.pone.0339293)
Supplement: S1 Table — (DOCX) [file pone.0339293.s001.docx]

**S1 Table. Pearson’s correlation between SV and EF in entire mouse cohort and in subgroups by sex.**

| **Group (# of Mice)** | **Pearson’s correlation (r) between SV and EF** | **p-value** |
| --- | --- | --- |
| Male (131) | 0.622 | 2.10×10^-15^ |
| Female (120) | 0.627 | 1.78×10^-14^ |
| All (251) | 0.526 | 3.07×10^-19^ |
