# Supplementary material for: Exercise mitigates high-fat diet-induced cardiac dysfunction via APOE genotype- and immune-dependent mechanisms: A photon-counting CT study in adult mice
Source: PLoS One. 2025 Dec 19;20(12):e0339293. doi: 10.1371/journal.pone.0339293 (PMC12716737; doi:10.1371/journal.pone.0339293)
Supplement: S2 Table — This group of 152 mice includes a subset of those in our cardiac PCCT study as well as mice that were not scanned with PCCT. Grouping in this table is only done by a single categorical variable at a time. (DOCX) [file pone.0339293.s002.docx]

**S2 Table. Mean and 95% confidence interval of distance run grouped by *APOE* genotype, *HN* status, and diet.** This group of 152 mice includes a subset of those in our cardiac PCCT study as well as mice that were not scanned with PCCT. Grouping in this table is only done by a single categorical variable at a time.

| **Category** | **Number of Mice** | **Mean Distance Run (km)** | **95% CI of Distance Run (km)** |
| --- | --- | --- | --- |
| *APOE2* | 51 | 39.11 | [35.08, 43.13] |
| *APOE3* | 53 | 27.70 | [23.78, 31.61] |
| *APOE4* | 48 | 32.99 | [28.33, 37.65] |
| *non-HN* | 80 | 30.49 | [27.22, 33.75] |
| *HN* | 72 | 36.21 | [32.47, 39.95] |
| CTRL | 94 | 34.31 | [31.63, 36.99] |
| HFD | 58 | 31.40 | [26.47, 36.33] |
