# Supplementary material for: Exercise mitigates high-fat diet-induced cardiac dysfunction via APOE genotype- and immune-dependent mechanisms: A photon-counting CT study in adult mice
Source: PLoS One. 2025 Dec 19;20(12):e0339293. doi: 10.1371/journal.pone.0339293 (PMC12716737; doi:10.1371/journal.pone.0339293)
Supplement: S3 Table — Lower and upper bounds of 95% confidence interval are shown in brackets below each mean value. (DOCX) [file pone.0339293.s003.docx]

**S3 Table. Mean and 95% confidence interval of physiological and cardiac metrics grouped by sex, *APOE* genotype, exercise regimen, and diet plan.** Lower and upper bounds of 95% confidence interval are shown in brackets below each mean value.

| **Sex** | **Genotype** | **Exercise?** | **Diet** | **Mass (g)** | **HR (bpm)** | **SV (mL)** | **EF (%)** | **RV SV (mL)** | **MM (mg)** |
| --- | --- | --- | --- | --- | --- | --- | --- | --- | --- |
| Female | *APOE2* | Yes | CTRL | 26.51  [25.58, 27.44] | 449.74  [426.83, 472.66] | 0.028 [0.025, 0.03] | 56.15  [53.33, 58.96] | 0.025  [0.023, 0.027] | 157.26  [147.34, 167.17] |
|  |  |  | HFD | 32.82  [29.12, 36.52] | 470.86  [442.61, 499.1] | 0.026 [0.021, 0.031] | 52.0  [45.58, 58.43] | 0.024  [0.019, 0.028] | 156.19  [145.84, 166.53] |
|  |  | No | CTRL | 25.93  [21.72, 30.14] | 464.57  [447.39, 481.76] | 0.025 [0.021, 0.03] | 60.89  [55.19, 66.58] | 0.023  [0.018, 0.028] | 141.34  [125.8, 156.87] |
|  |  |  | HFD | 41.01  [31.39, 50.64] | 442.78  [401.97, 483.58] | 0.031 [0.022, 0.04] | 53.05  [44.38, 61.73] | 0.03  [0.02, 0.04] | 189.81  [146.21, 233.41] |
|  | *APOE3* | Yes | CTRL | 27.89  [26.64, 29.13] | 470.8  [447.27, 494.33] | 0.024 [0.021, 0.028] | 52.78  [45.46, 60.1] | 0.023  [0.019, 0.027] | 153.78  [136.54, 171.02] |
|  |  |  | HFD | 33.26  [30.72, 35.8] | 472.29  [446.79, 497.78] | 0.027 [0.024, 0.03] | 54.11  [48.37, 59.84] | 0.025  [0.022, 0.027] | 154.24  [139.5, 168.97] |
|  |  | No | CTRL | 29.44  [28.11, 30.76] | 448.82  [425.41, 472.23] | 0.021 [0.017, 0.025] | 45.65  [37.23, 54.07] | 0.018  [0.014, 0.023] | 156.8  [144.17, 169.43] |
|  |  |  | HFD | 49.66  [44.2, 55.12] | 442.97  [418.88, 467.06] | 0.027 [0.022, 0.033] | 47.12  [38.66, 55.57] | 0.025  [0.021, 0.03] | 195.84  [177.01, 214.67] |
|  | *APOE4* | Yes | CTRL | 26.27  [23.05, 29.48] | 489.5  [472.23, 506.77] | 0.024 [0.022, 0.026] | 58.19  [54.62, 61.76] | 0.022  [0.02, 0.023] | 150.88  [140.13, 161.62] |
|  |  |  | HFD | 35.45  [32.29, 38.61] | 513.51  [485.85, 541.18] | 0.031 [0.026, 0.035] | 62.09  [58.99, 65.18] | 0.029  [0.025, 0.033] | 165.95  [155.42, 176.47] |
|  |  | No | CTRL | 28.4  [25.84, 30.96] | 455.76  [407.95, 503.56] | 0.018 [0.012, 0.023] | 44.51  [32.25, 56.77] | 0.019  [0.014, 0.024] | 154.23  [140.32, 168.13] |
|  |  |  | HFD | 45.97  [38.27, 53.68] | 421.47  [371.62, 471.32] | 0.026 [0.022, 0.03] | 49.52  [41.46, 57.59] | 0.024  [0.02, 0.029] | 185.38  [146.75, 224.01] |
| Male | *APOE2* | Yes | CTRL | 31.47  [30.75, 32.18] | 468.57  [445.2, 491.95] | 0.033 [0.029, 0.036] | 55.1  [51.93, 58.28] | 0.03  [0.026, 0.033] | 197.44  [176.44, 218.45] |
|  |  |  | HFD | 44.19  [40.03, 48.35] | 463.48  [431.89, 495.07] | 0.038 [0.032, 0.044] | 51.79  [46.09, 57.5] | 0.035  [0.029, 0.041] | 208.08  [185.59, 230.58] |
|  |  | No | CTRL | 31.68  [30.37, 32.99] | 470.86  [433.49, 508.23] | 0.026 [0.021, 0.03] | 47.11  [38.1, 56.11] | 0.025  [0.02, 0.03] | 203.12  [176.08, 230.16] |
|  |  |  | HFD | 44.71  [39.29, 50.13] | 440.68  [386.77, 494.58] | 0.027 [0.02, 0.034] | 40.1  [31.81, 48.38] | 0.026  [0.019, 0.034] | 240.12  [207.58, 272.67] |
|  | *APOE3* | Yes | CTRL | 34.13  [33.16, 35.1] | 464.79  [440.58, 489.0] | 0.033 [0.03, 0.036] | 51.99  [48.91, 55.07] | 0.03  [0.027, 0.032] | 197.43  [185.22, 209.65] |
|  |  |  | HFD | 42.57  [37.78, 47.35] | 447.52  [425.13, 469.92] | 0.023 [0.018, 0.029] | 41.86  [34.94, 48.78] | 0.023  [0.017, 0.029] | 180.05  [160.19, 199.91] |
|  |  | No | CTRL | 33.75  [28.1, 39.4] | 430.43  [414.21, 446.64] | 0.023 [0.021, 0.026] | 39.75  [30.76, 48.74] | 0.021  [0.019, 0.024] | 188.45  [159.79, 217.1] |
|  |  |  | HFD | 47.85  [44.06, 51.64] | 445.96  [425.09, 466.83] | 0.024 [0.02, 0.028] | 41.14  [35.17, 47.1] | 0.023  [0.018, 0.027] | 206.24  [191.9, 220.58] |
|  | *APOE4* | Yes | CTRL | 30.75  [29.32, 32.18] | 483.29  [467.24, 499.33] | 0.033 [0.028, 0.037] | 52.4  [47.32, 57.47] | 0.029  [0.025, 0.032] | 202.2  [178.8, 225.61] |
|  |  |  | HFD | 35.94  [28.71, 43.18] | 466.65  [400.72, 532.59] | 0.032 [0.022, 0.041] | 47.54  [38.37, 56.71] | 0.03  [0.021, 0.04] | 204.08  [151.65, 256.52] |
|  |  | No | CTRL | 26.64  [22.8, 30.48] | 448.71  [429.85, 467.58] | 0.02 [0.017, 0.023] | 44.51  [39.12, 49.9] | 0.018  [0.014, 0.021] | 151.78  [131.82, 171.74] |
|  |  |  | HFD | 47.6  [43.53, 51.67] | 432.86  [397.84, 467.87] | 0.028 [0.023, 0.033] | 46.19  [38.31, 54.07] | 0.027  [0.022, 0.032] | 219.16  [190.55, 247.77] |
