# Supplementary material for: Exercise mitigates high-fat diet-induced cardiac dysfunction via APOE genotype- and immune-dependent mechanisms: A photon-counting CT study in adult mice
Source: PLoS One. 2025 Dec 19;20(12):e0339293. doi: 10.1371/journal.pone.0339293 (PMC12716737; doi:10.1371/journal.pone.0339293)
Supplement: S4 Table — (DOCX) [file pone.0339293.s004.docx]

**S4 Table. Summary of significant effects in our GLMs that did not involve exercise or diet.**

| **Metric** | **Predictor** | **Coefficient (β)** | **Corrected p-value** | **Interpretation** |
| --- | --- | --- | --- | --- |
| Mass | Geno3[T.APOE2] | -0.135 | 0.0235 | *APOE2* genotype has a significant negative effect on body mass relative to *APOE3*. |
| Mass | Geno3[T.APOE4] | -0.161 | 4.96×10^-3^ | *APOE4* genotype has a significant negative effect on body mass relative to *APOE3*. |
| Mass | Sex[T.Female] | -0.109 | 0.0306 | Female sex has a significant negative effect on body mass relative to male sex. |
| Mass | Age | 8.674×10^-3^ | 0.0101 | Aging has a significant positive effect on body mass. |
| Stroke Volume | Age | 5.34×10^-4^ | 2.00×10^-3^ | Aging has a significant positive effect on stroke volume. |
| RV Stroke Volume | HN[T.HN] | -5.68×10^-3^ | 0.0253 | *HN* has a significant negative effect on RV stroke volume relative to *non-HN*. |
| RV Stroke Volume | Age | 4.73×10^-4^ | 3.50×10^-3^ | Aging has a significant positive effect on RV stroke volume. |
| Myocardial Mass | Sex[T.Female] | -0.173 | 2.56×10^-3^ | Female sex has a significant negative effect on myocardial mass relative to male sex. |
| Myocardial Mass | Geno3[T.APOE4]:  HN[T.HN] | -0.150 | 0.0105 | The combination of *APOE4* genotype and *HN* has a significant negative effect on myocardial mass. |
| Myocardial Mass | Age | 0.0176 | 1.09×10^-5^ | Aging has a significant positive effect on myocardial mass. |
