# Supplementary material for: Exercise mitigates high-fat diet-induced cardiac dysfunction via APOE genotype- and immune-dependent mechanisms: A photon-counting CT study in adult mice
Source: PLoS One. 2025 Dec 19;20(12):e0339293. doi: 10.1371/journal.pone.0339293 (PMC12716737; doi:10.1371/journal.pone.0339293)
Supplement: S5 Table — For each significant predictor, we report the value of two test statistics (Wilks’ λ and Pillai’s trace), with the associated p-value shown in parentheses. (DOCX) [file pone.0339293.s005.docx]

**S5 Table. Summary of significant effects in our MANOVA with stroke volume and ejection fraction as dependent variables.** For each significant predictor, we report the value of two test statistics (Wilks’ λ and Pillai’s trace), with the associated p-value shown in parentheses.

| **Predictor** | **Wilks’ λ (p-value)** | **Pillai’s Trace (p-value)** |
| --- | --- | --- |
| Exercise | 0.9648 (0.0168) | 0.0352 (0.0168) |
| Genotype:Diet | 0.9371 (0.0051) | 0.0634 (0.0052) |
| Sex:Exercise | 0.9493 (0.0027) | 0.0507 (0.0027) |
| HN:Diet | 0.9623 (0.0124) | 0.0377 (0.0124) |
| Age | 0.9222 (0.0001) | 0.0778 (0.0001) |
