# Supplementary figures and images for: Exercise mitigates high-fat diet-induced cardiac dysfunction via APOE genotype- and immune-dependent mechanisms: A photon-counting CT study in adult mice
Source: PLoS One. 2025 Dec 19;20(12):e0339293. doi: 10.1371/journal.pone.0339293 (PMC12716737; doi:10.1371/journal.pone.0339293)

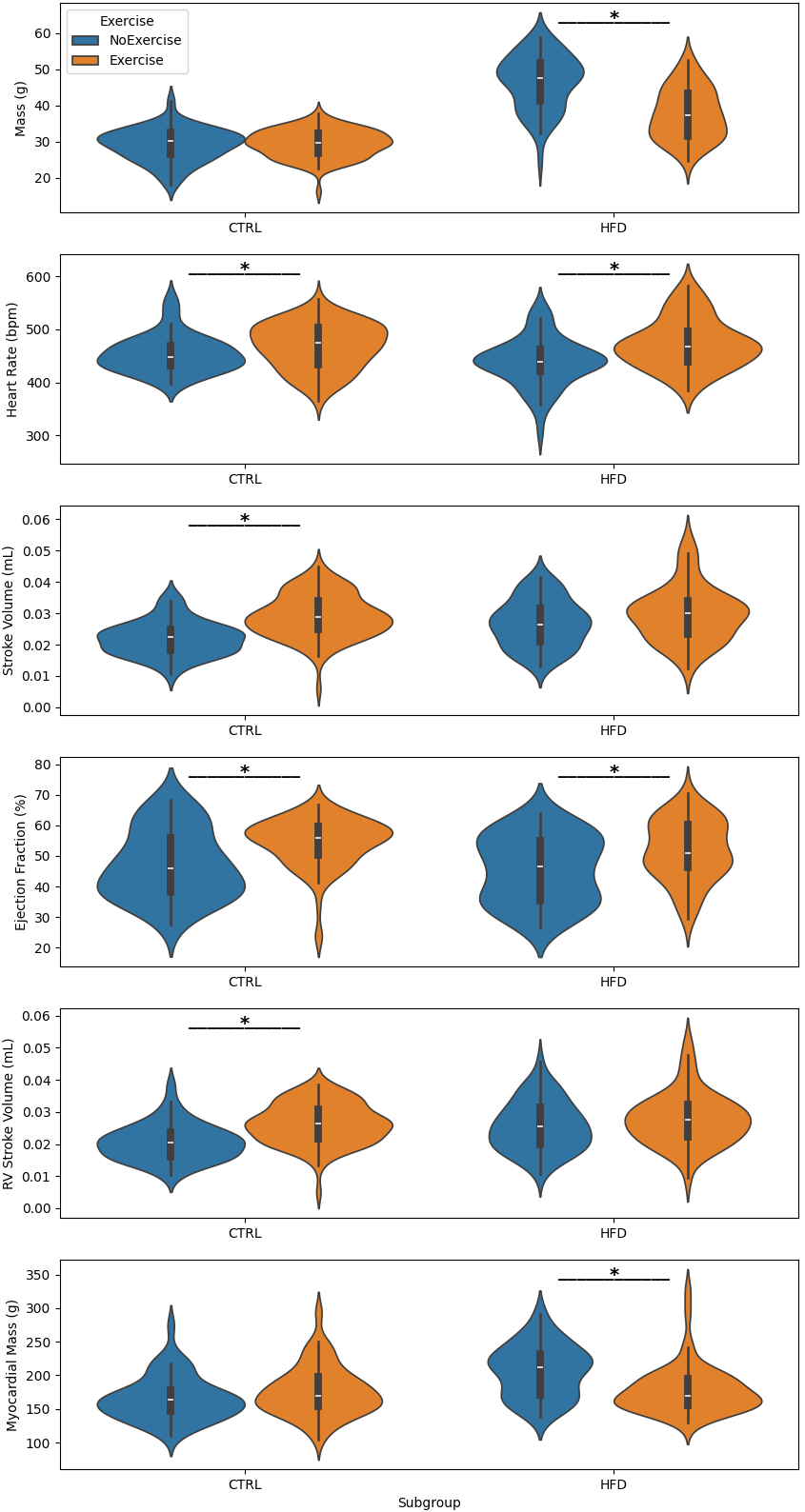

Supplement: S1 Fig — Violin plots show six physiological and cardiac metrics stratified by diet (CTRL vs. HFD) and exercise status. Boxplots within violins show the median and interquartile range. Asterisks denote significant differences (p < 0.05, Mann-Whitney U test) by exercise status within a diet subgroup. (TIF) [file pone.0339293.s006.tif]

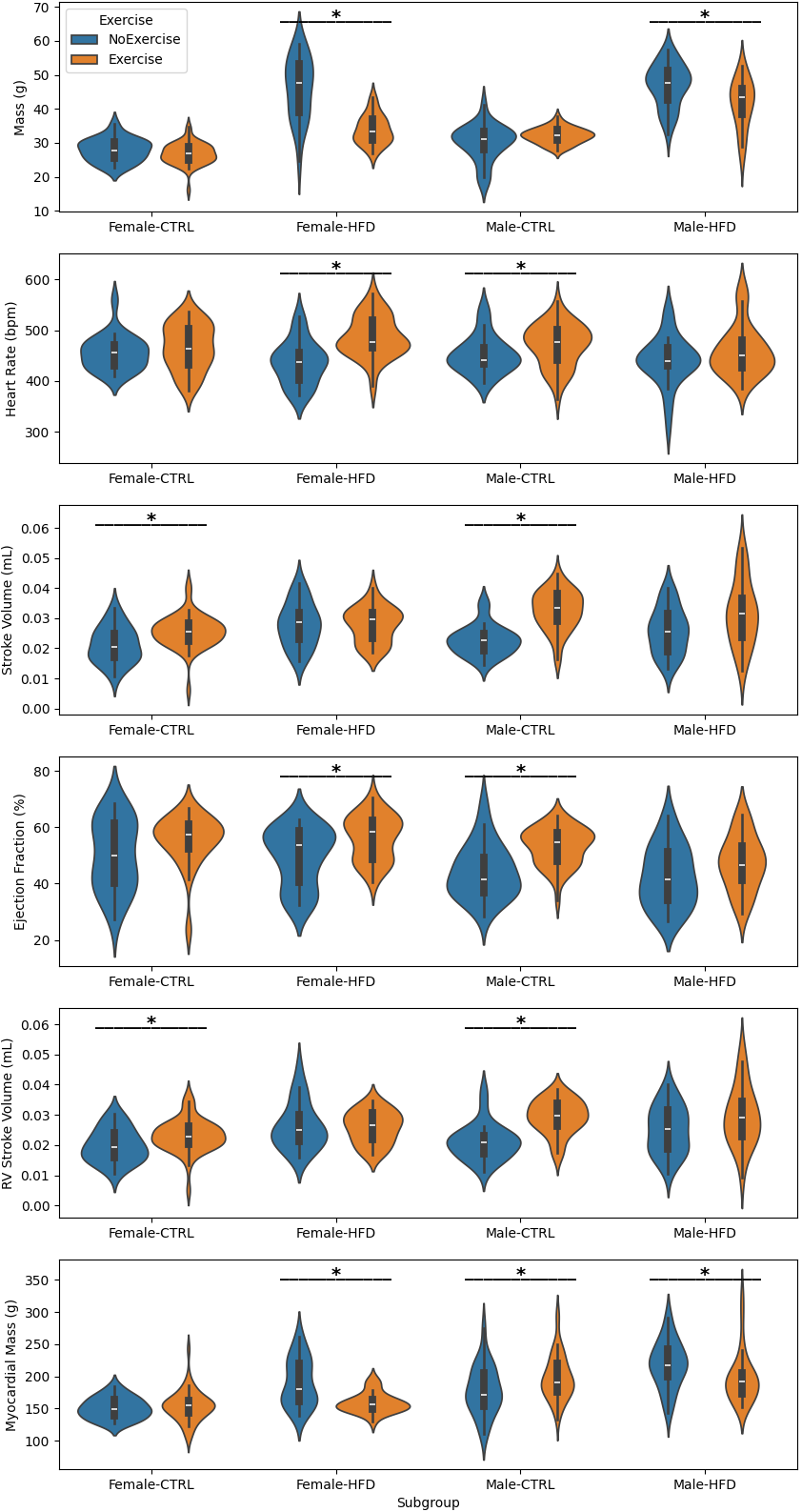

Supplement: S2 Fig — Violin plots show six physiological and cardiac metrics stratified by sex (female/male), diet (CTRL/HFD), and exercise status. Boxplots within violins show the median and interquartile range. Asterisks denote significant differences (p < 0.05, Mann-Whitney U test) by exercise status within a sex-diet subgroup. (TIF) [file pone.0339293.s007.tif]
